# Supplementary material for: Insights on Aggregation of Hen Egg-White Lysozyme from Raman Spectroscopy and MD Simulations
Source: Molecules. 2022 Oct 21;27(20):7122. doi: 10.3390/molecules27207122 (PMC9609503; doi:10.3390/molecules27207122)
Supplement: Supplementary file 1 [file molecules-27-07122-s001.zip › molecules-1962403-supplementary.pdf]

molecules

MDPI

Article

Insights on Aggregation of Hen Egg-White Lysozyme from Raman Spectroscopy and MD Simulations

Divya Chalapathi [1], Amrendra Kumar [2], Pratik Behera [3], Shijulal Nelson Sathi [3], Rajaram Swaminathan [2,*] and Chandrabhas Narayana [1,*]

1 Chemistry and Physics of Materials Unit, School of Advanced Materials, Jawaharlal Nehru Centre for Advanced Scientific Research, Bengaluru 560064, India
2 Department of Bioscience and Bioengineering, Indian Institute of Technology-Guwahati, North Amingaon, Guwahati 781039, India
3 Transdisciplinary Biology Program, Rajiv Gandhi Centre for Biotechnology, Thycaud Post, Poojapura, Thiruvananthapuram 695014, India
* Correspondence: rsw@iitg.ac.in (R.S.); cbhas@jncasr.ac.in or cbhas@rgcb.res.in (C.N.); Tel.: +91-471-2347-973 (R.S. & C.N.)

Abstract: Protein misfolding and aggregation play a significant role in several neurodegenerative diseases. In the present work, the spontaneous aggregation of hen egg-white lysozyme (HEWL) in an alkaline pH 12.2 at an ambient temperature was studied to obtain molecular insights. The time-dependent changes in spectral peaks indicated the formation of β sheets and their effects on the backbone and amino acids during the aggregation process. Introducing iodoacetamide revealed the crucial role of intermolecular disulphide bonds amidst monomers in the aggregation process. These findings were corroborated by Molecular Dynamics (MD) simulations and protein-docking studies. MD simulations helped establish and visualize the unfolding of the proteins when exposed to an alkaline pH. Protein docking revealed a preferential dimer formation between the HEWL monomers at pH 12.2 compared with the neutral pH. The combination of Raman spectroscopy and MD simulations is a powerful tool to study protein aggregation mechanisms.

Keywords: protein aggregation; Raman spectroscopy; molecular dynamics; protein–protein interactions; structural biology

Citation: Chalapathi, D.; Kumar, A.; Behera, P.; Sathi, S.N.; Swaminathan, R.; Narayana, C. Insights on Aggregation of Hen Egg-White Lysozyme from Raman Spectroscopy and MD Simulations. *Molecules* **2022**, *27*, 7122. https://doi.org/10.3390/molecules27207122

Academic Editor: Ivo Piantanida

Received: 25 September 2022
Accepted: 15 October 2022
Published: 21 October 2022

Publisher's Note: MDPI stays neutral with regard to jurisdictional claims in published maps and institutional affiliations.

Copyright: © 2022 by the authors. Licensee MDPI, Basel, Switzerland. This article is an open access article distributed under the terms and conditions of the Creative Commons Attribution (CC BY) license (https://creativecommons.org/licenses/by/4.0/).

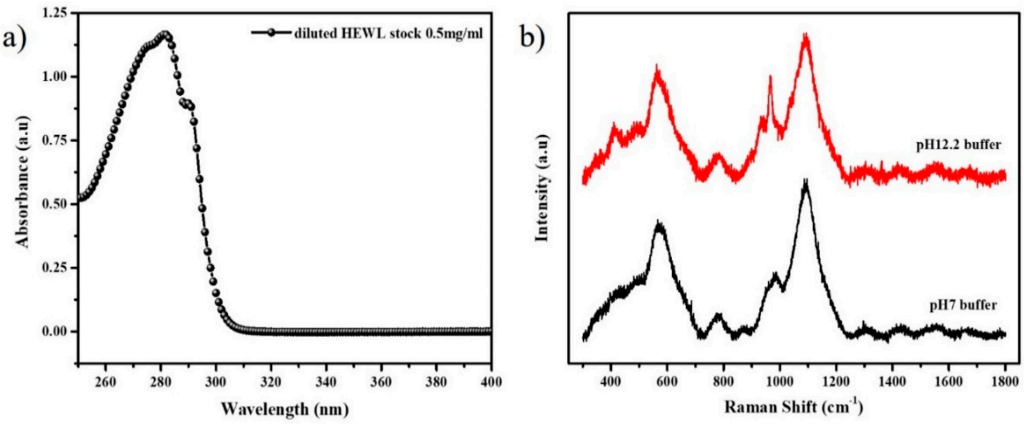

Figure S1. a) UV-visible absorption spectra of 10 times diluted HEWL stock from 250 nm to 400 nm used to calculate the protein concentration in the stock solution, b) Raman spectra of the pH 7.0 and pH 12.2 buffer using 532 nm laser.

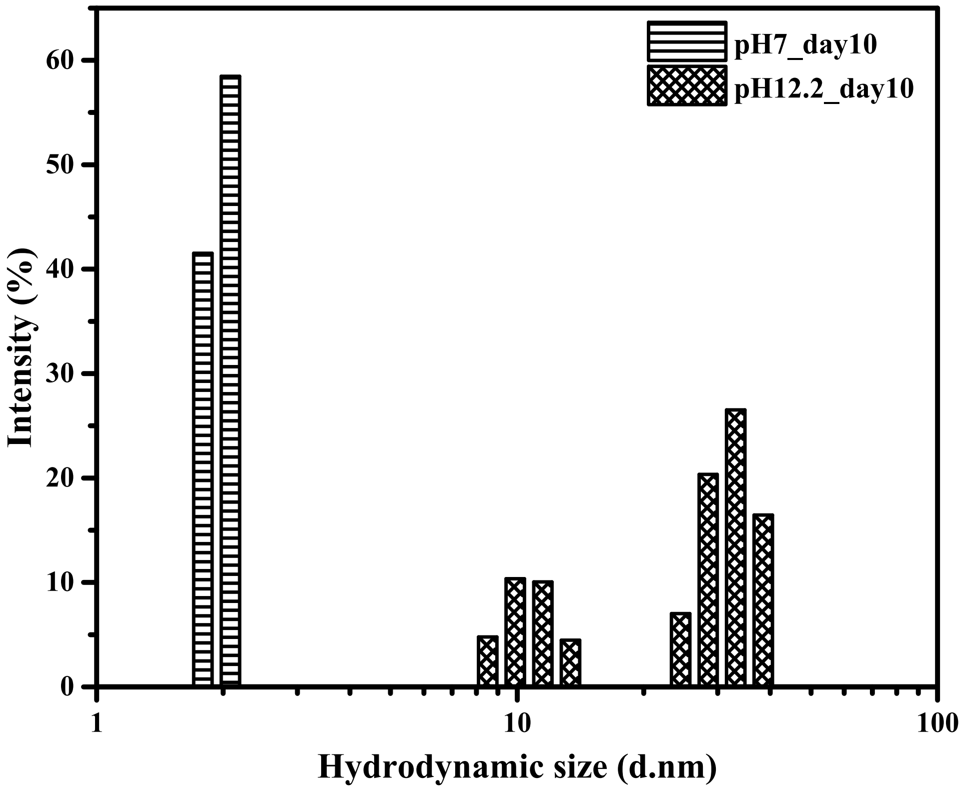

Figure S2. DLS spectra of the protein samples at 240 hr in pH 7.0 (control) and pH 12.2 (experiment) buffers.

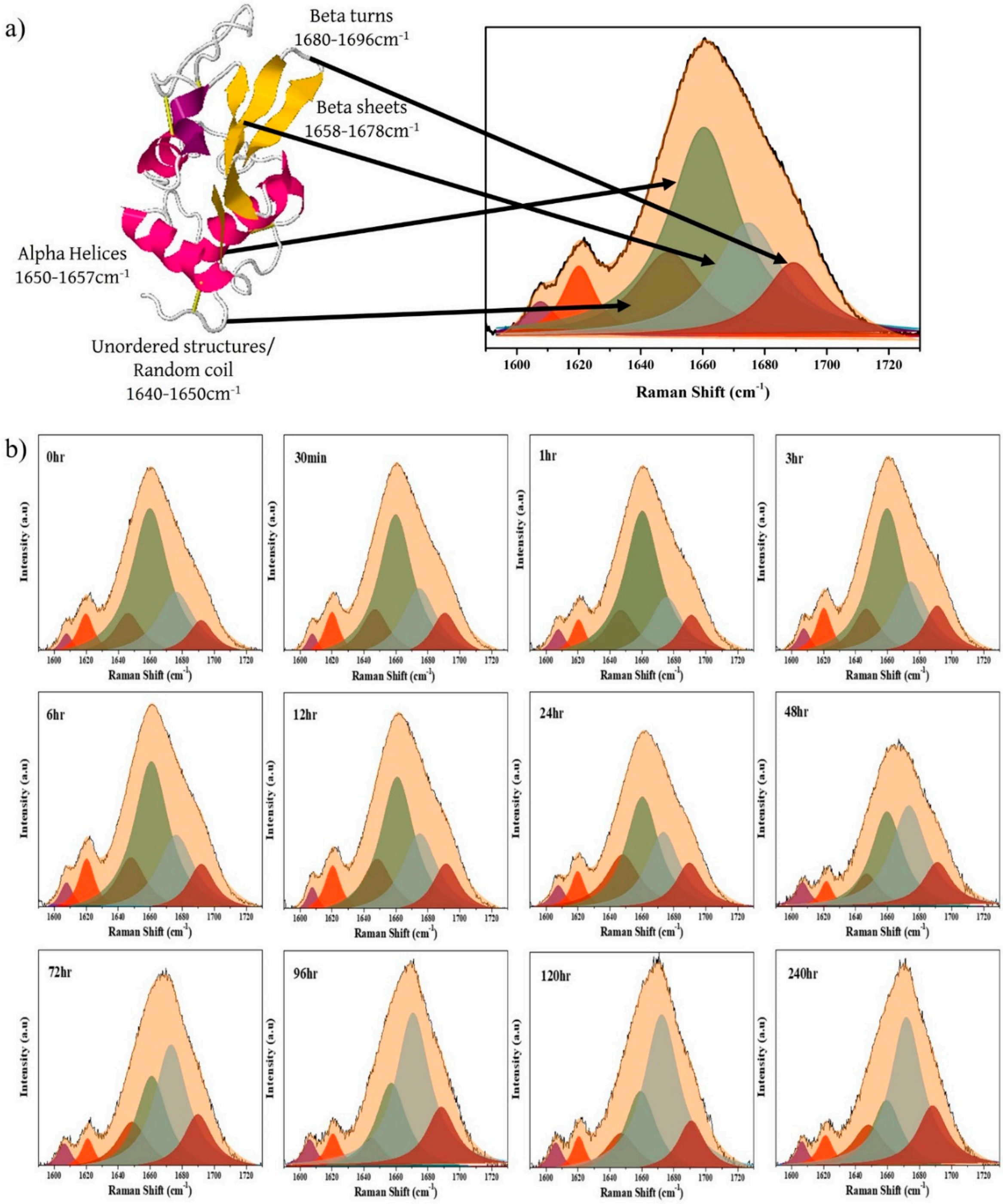

Figure S3. a) Deconvolution of the Amide I band into various secondary structure components, b) Deconvolution of the Amide I band at various time points, for the protein in pH 12.2. This shows a gradual decrease in alpha-helical content and an increase in beta-sheet content after 12 hr of incubation of the protein in pH 12.2.

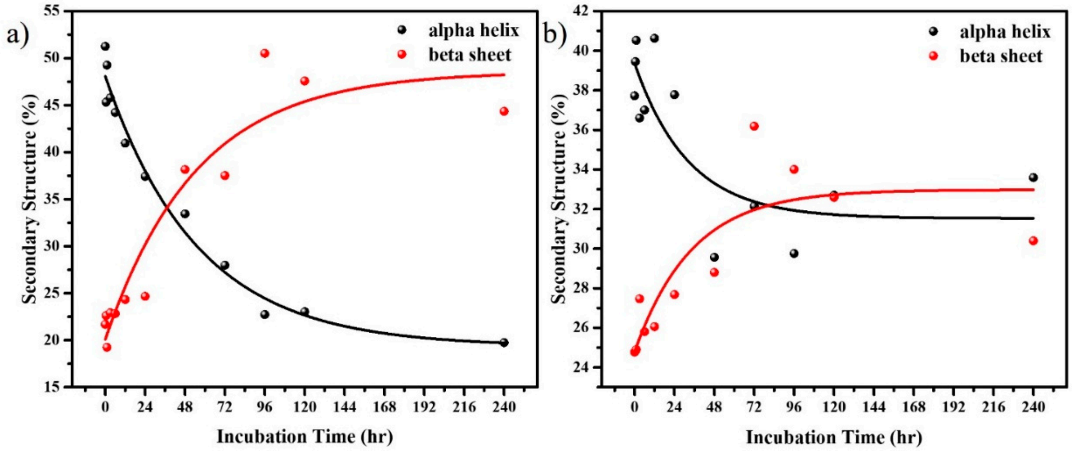

Figure S4. The percentage of the alpha-helix and the beta-sheet secondary structure was determined from the analysis of the Amide I Raman band for the HEWL incubated in pH 12.2 buffer, at two different concentrations, a) 120 μM and b) 30 μM.

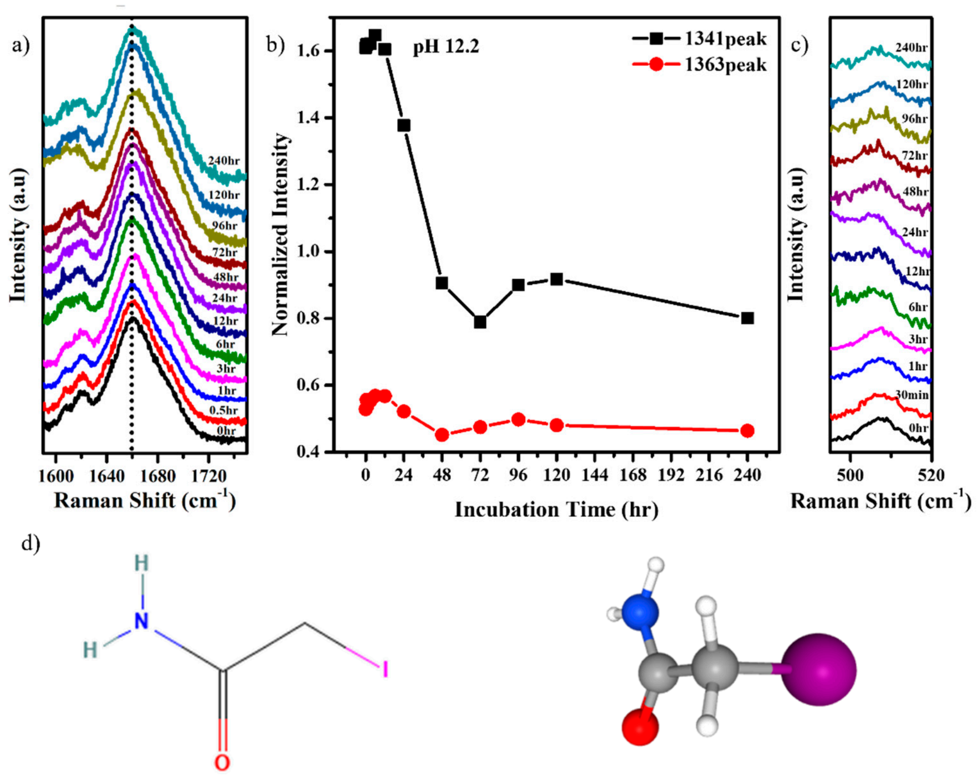

Figure S5. a) Stack plot of the Amide I band of the HEWL protein in pH 7.0 buffer, in the presence of idoacetamide, b)Intensities of the 1341 and 1363 cm^{-1} Raman peaks corresponding to the Tryptophan residues for the protein in pH 12.2. Their ratio acts as a hydrophobicity marker, and c) Stack plot of 505cm^{-1} Raman peaks for the HEWL in pH 12.2 buffer, in the presence of iodoacetamide, an aggregation-inhibiting small molecule and d) molecular structure of iodoacetamide.

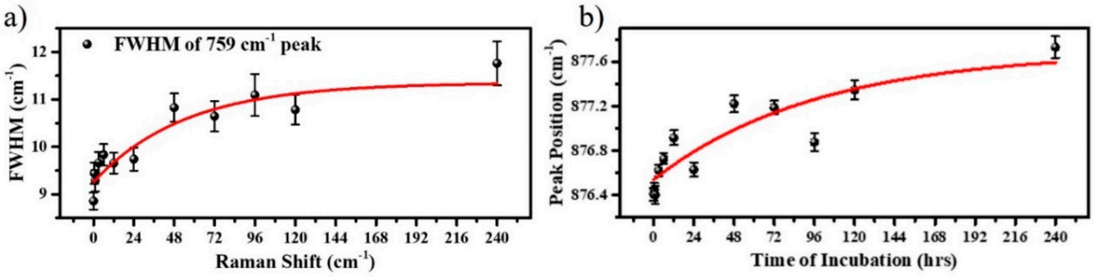

Figure S6. a) FWHM of 759 cm^{-1} Raman peak and b) Position of the Raman peak around 876 cm^{-1} as a function of time for the protein in pH 12.2.

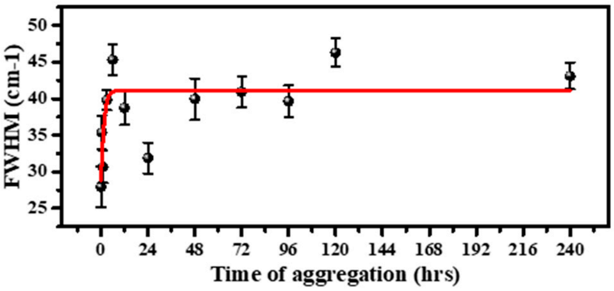

Figure S7. FWHM of the 1446cm^{-1} Raman peak for the protein in pH 12.2 suddenly increases from 0 to 6 hr, validating that the protein unfolded when placed in an alkaline buffer.

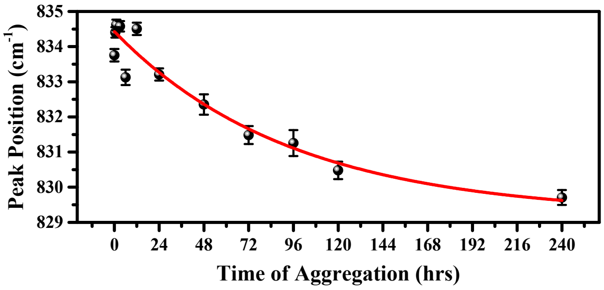

Figure S8. Variation in the position of the Raman peak around 830cm^{-1} for the protein at various time points of aggregation in pH 12.2.

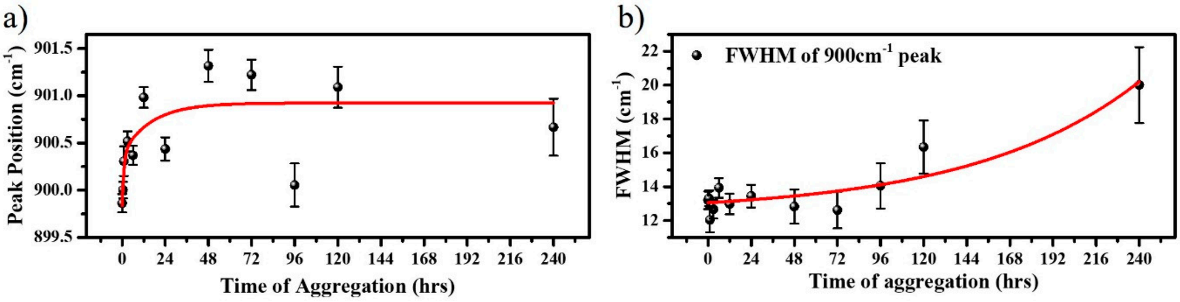

Figure S9. a) Peak position and b) FWHM, of the peak around 900 cm^{-1} of the protein at various time points of aggregation in pH 12.2.

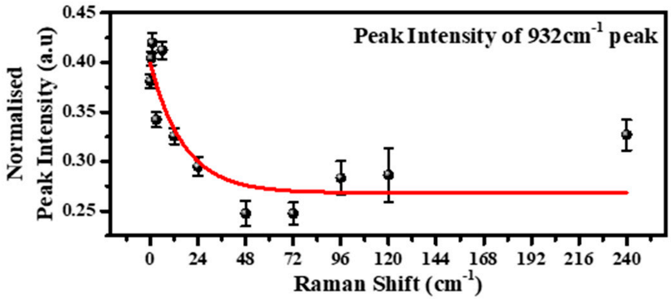

Figure S10. Change in the intensity of the Raman peak around 932 cm^{-1} for the protein in pH 12.2.

Molecular Dynamics Simulations:

Protein Unfolding:

To correlate to the experimental conditions of the HEWL protein in pH 7.0 and pH 12.2 environments, the following systems were chosen for study, as classical MD simulations don't allow for the breaking of covalent bonds during a simulation.

N – protein in pH 7.0 with the disulphide bonds intact;

NBB – protein in pH 7.0 with the disulphide bonds broken;

H – protein in pH 12.2 with the disulphide bonds intact;

HBB – protein in pH 12.2 with the disulphide bonds broken;

Various experimental studies prove that when a protein is placed in an alkaline pH environment, the disulphide bonds no longer remain intact and break to form dangling -S-H bonds. While in a neutral pH environment, the bonds remain unbroken, thus keeping the system intact. So, it was the most logical choice to choose the systems N and HBB as the systems under study to compare with the experiments performed in this paper. To clarify the behaviour in all possible and hypothetical situations, we conducted the MD simulations for all 4 cases of N, NBB, H and HBB. The following are the results corresponding to the same,

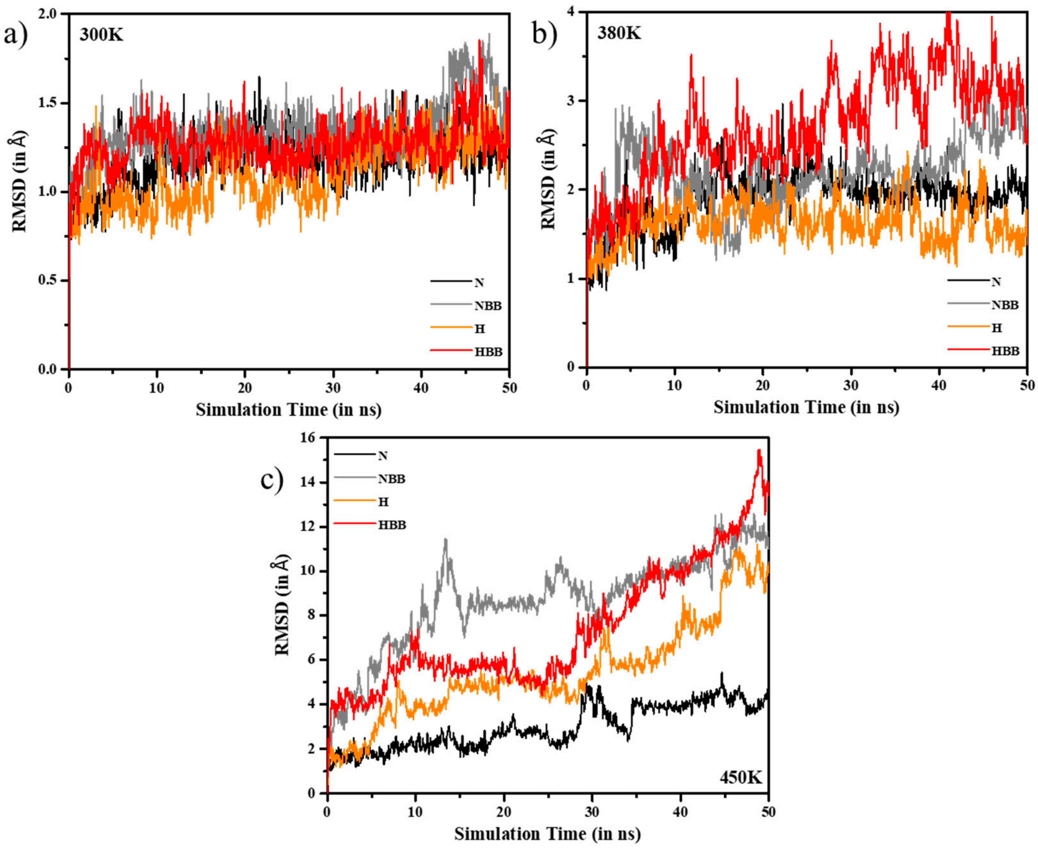

Figure SA. Deviations in the trajectory of the simulation for the protein in all variations of N, NBB, H and HBB, conducted at a) 300K, b) 380K and, c) 450K.

The aim of increasing the temperature of the simulation was to hasten the unfolding process, as it would be impractical to conduct simulation at room temperature for time scales of a few hours. From these plots in Fig. SA, we see that the temperatures of 300K and 380K are insufficient to unfold the protein, while at 450K, the protein begins to unfold well within 50ns. Though N also has a high value of RMSD through the simulation, it is experimentally not feasible and hence is ruled out.

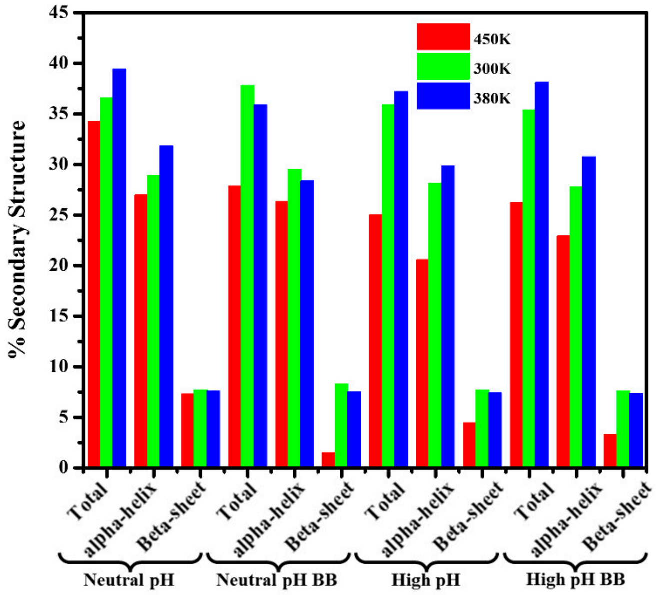

Figure SB. Secondary structure elements of the protein after 50 ns simulation at various temperatures. Red (450 K), Green (300 K) and Blue (380 K). At 450K, there is a maximum change in the secondary structure, indicating that the protein has partially unfolded. There is a higher reduction in the secondary structure content at pH 12.2 than at pH 7.0.

The simulation results show that H has a higher deviation in the trajectory while N remains stable. While in the case of NBB and HBB, HBB shows a more significant deflection. Thus from both Fig. A and Fig. B, we can confidently claim that an alkaline environment that causes the unfolding of the protein can be replicated by using 450K environment temperature and HBB, with the disulphide bonds broken.

Protein Aggregation:

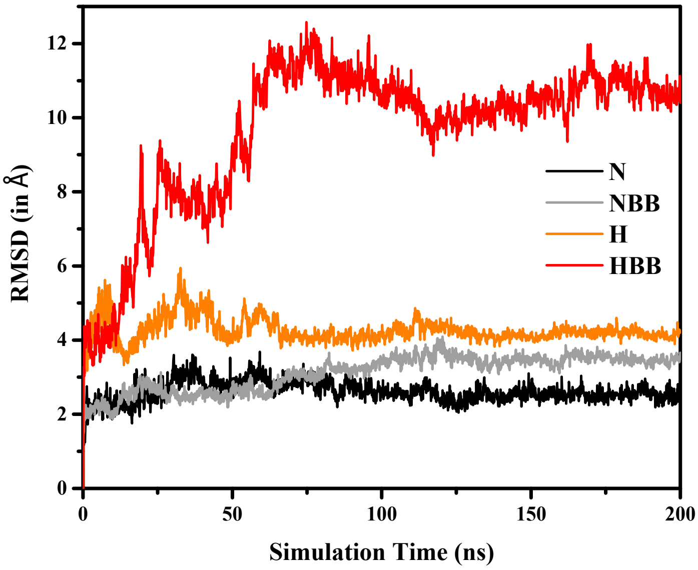

Figure S11. The stability of the protein is studied using the RMSD vs simulation time plot. It is observed that the deviation in the RMSD of HBB is quite significant as compared to the N, H, and NBB. This can be attributed to the untangled N terminal dangling in the solvent.

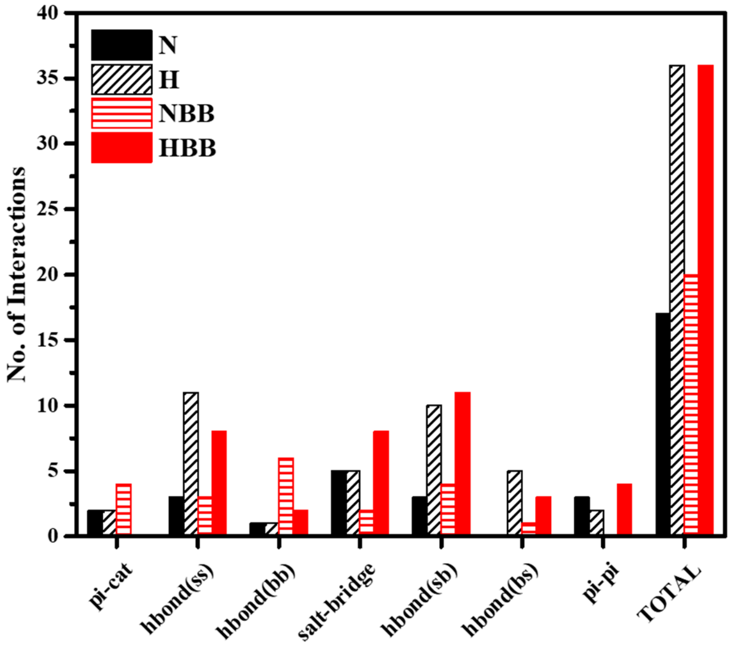

Figure SC. Interactions among the homodimer aggregates are represented by N (solid, black), H (striped, black), NBB (striped, red) and HBB (solid, red). The types of interaction represented from left to right are: 1) pi – cation, 2) Hydrogen bond (side chain-side chain), 3) Hydrogen bond (backbone-backbone), 4) Salt bridges, 5) Hydrogen bond (sidechain-backbone), 6) Hydrogen bond (backbone-sidechain), 7) Pi-Pi and 8) Total interactions. pH 12.2 contributes to a higher no. of interactions than pH 7.0.

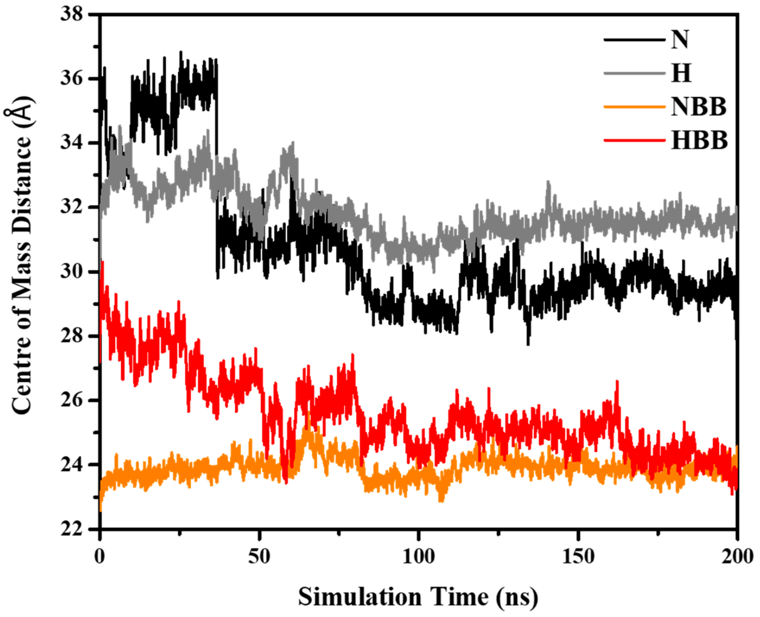

Figure SD. The distance of the Center of mass between the homodimer aggregate is plotted as a function of simulation time for N (Black), NBB (Grey), H (Orange) and HBB (Red). H and NBB remain stable from the start to the end of the simulation.

In N, there is an initial transition around 50 ns, after which the dimer stabilises at 30 Å. While in HBB, the initial docking distance started at 30 Å, it decreased through the

simulation and continued to decrease till the end of it. This is valid proof that the chains come closer to forming a more stable aggregate.

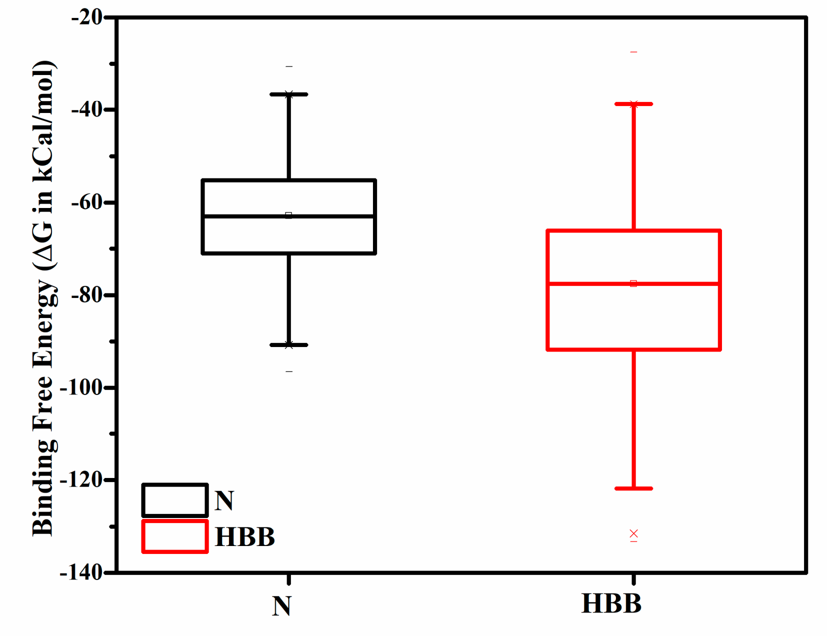

Figure S12. Binding free energy of the docked proteins with the monomers N and HBB;

M-GBSA allows one to computationally calculate the protein-protein binding energy though it has limited accuracies, especially with the charged systems.[1] From the above plot, it is clear that HBB has lower energy than N and H systems at -77 kCal/mol. It is interesting to note that the value is higher than that of the NBB system. This can be explained by Fig. S13 & SD, that the NBB is a very stable structure. From Fig. SC & SD, it is clear that the HBB system is the most preferred for aggregation. Still, an increased RMSD (seen in Fig. S11) indicates free dangling N terminals, which destabilize the system and thus eventually result in a slightly higher free energy value (as seen in Fig. SE).

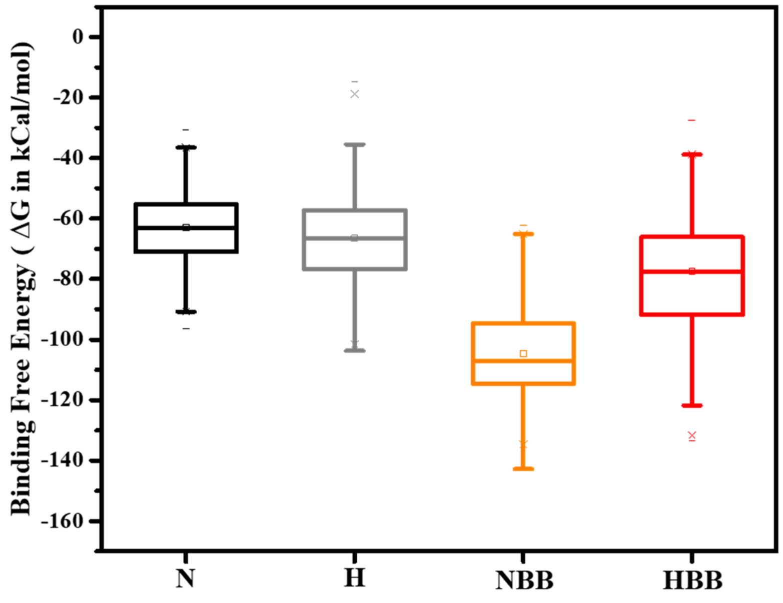

Figure SE. Binding free energy was calculated using the frames of the last 25ns of the simulation using MM-GBSA for the dimers of N (black), H (grey), NBB (orange) and HBB (red).

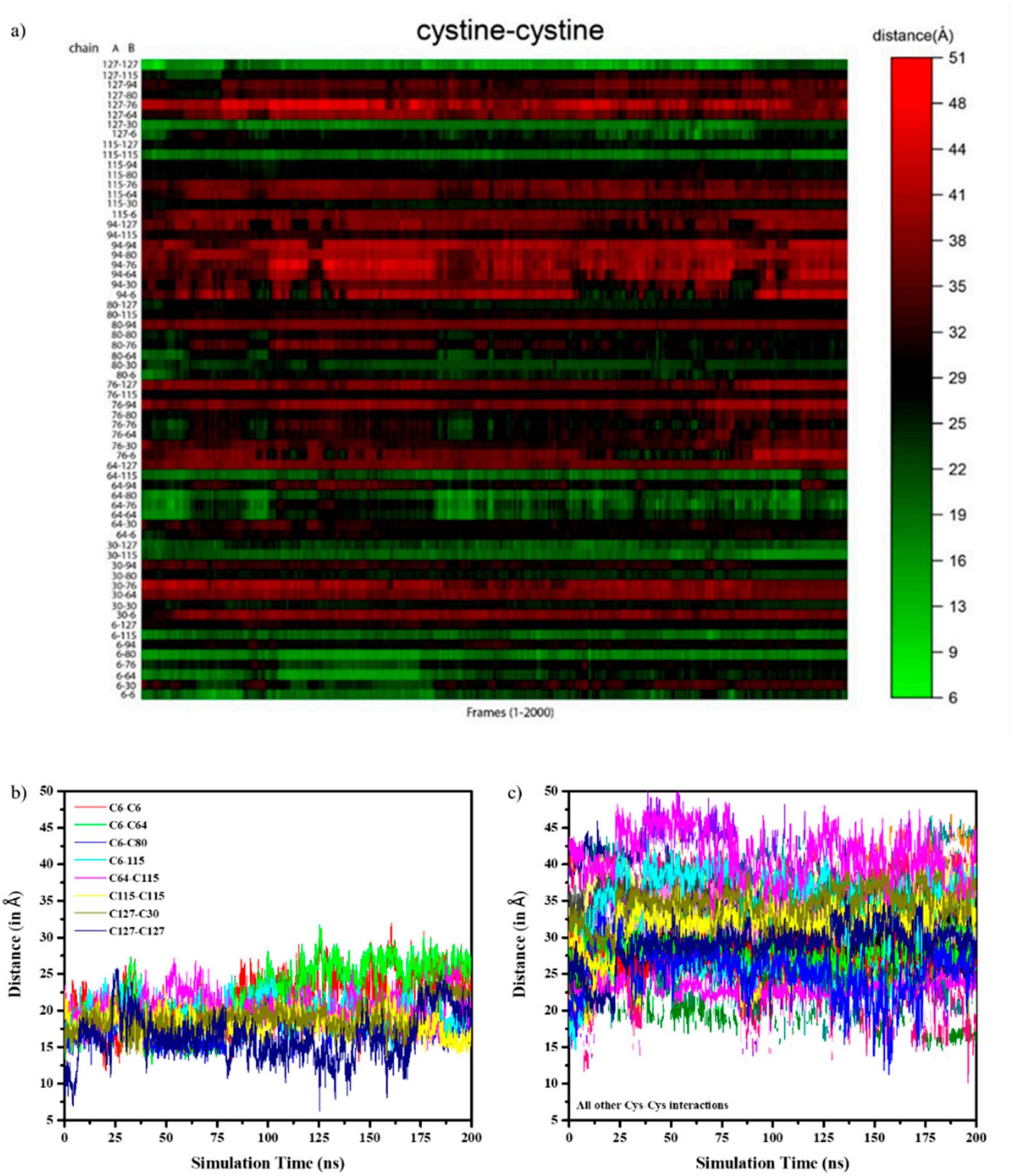

Figure S13. a) Heat map representing all the various cysteine -cysteine distance residues. The distance map from 6 Å (green) representing the closeness of the cysteines from 2 different monomers to 51 Å (red), representing the farther away cysteines, can be used to identify the Cysteine residues that come close. b) The line plots corresponding to the closest occurring cysteine residues in the course of the 200 ns of the simulation, c) The line plots corresponding to every other cysteine residue's interaction during the duration of the simulation.

Eight cysteine residues in each monomeric unit allow for 64 possible interactions in the dimeric aggregate. If Cysteine residues got close enough, a new disulphide could be established amidst the monomers, resulting in a covalently bonded dimeric state. Hence a heat map was convenient to monitor their distances throughout the 200 ns of the simulation, as seen in the following plot, along with a few selected possible combinations represented by a line map. (Fig. S13)

Table S1. Peak assignments to the Raman modes obtained for HEWL

PEAK (cm^{-1})	RAMAN MODE	REFERENCE
505	S-S stretch (gauche–gauche–gauche) in cysteine	[2,3]
525	S-S stretch (gauche–gauche–trans) in cysteine	[2,3]
575	Tryptophan	[4]
623	C-C twisting mode in Phenylalanine	[4]
643	C-C twisting mode of tyrosine	[5]
695	C-S bond	[6]
720	C-S stretch trans conformation	[7]
759	Tryptophan – Coupled vibrations of in-phase breathings of benzene and pyrrole (indole ring)	[8]
833	Fermi resonance of ring fundamental and overtone - Tyrosine	[7]
853	Fermi resonance of ring fundamental and overtone - Tyrosine	[4]
877	Tryptophan- benzene ring + N1H motion	[9]
900	N-C_{α}-C stretch of skeleton	[6]
930	N-C_{α}-C stretch of skeleton	[6,9]
1004	Ring breathing of benzene in Phenylalanine	[8]
1011	Tryptophan ring breathing	[4]
1076	CH_2 symmetric rock+ C_{α}-C stretching	[6]
1105	CH_2 symmetric rock+ C_{α}-C stretching	[6]
1128	CH_2 symmetric rock+ C_{α}-C stretching	[6]
1236	Type II β-turn strand, amide III	[6]
1255	Amide III	[4]
1300	CH_2- twisting, wagging	[4]
1339 & 1362	Tryptophan Fermi resonances between the fundamental in-plane N_1=C_8 stretching and combination bands of ring out-of-plane deformations	[8]
1426	Pyrrole [ν(N1–C2=C3) + δ(NH)] + benzene δ(CH)	[9]
1448	CH_2, CH_3 and CH deformation and scissoring	[6]
1458	CH_2, CH_3 and CH deformation and scissoring	[6]
1554	C=C Tryptophan	[4,9]
1580	Tryptophan, amide II, Tyrosine	[4]
1607	Tyr, Phe ring vibration; C=C phenylalanine, tyrosine	[4]
1621	ν_s(ring) + δ (OH) - Tryptophan	[4,6,9]
1661	Amide I band	[6,7]

References

1. Wang, J.; Hou, T.; Xu, X. Recent Advances in Free Energy Calculations with a Combination of Molecular Mechanics and Continuum Models. *Current Computer-Aided Drug Design* **2006**, *2*, 287-306, doi:http://dx.doi.org/10.2174/157340906778226454.
2. Kurouski, D.; Van Duyne, R.P.; Lednev, I.K. Exploring the structure and formation mechanism of amyloid fibrils by Raman spectroscopy: a review. *Analyst* **2015**, *140*, 4967-4980, doi:10.1039/C5AN00342C.
3. Teizo Kitagawa, S.H. Raman Spectroscopy of Proteins. In *Handbook of Vibrational Spectroscopy*, Peter Griffiths, J.M.C., Ed.; John Wiley & Sons, Ltd: 2006.
4. Talari, A.C.S.; Movasaghi, Z.; Rehman, S.; Rehman, I.u. Raman Spectroscopy of Biological Tissues. *Applied Spectroscopy Reviews* **2015**, *50*, 46-111, doi:10.1080/05704928.2014.923902.
5. Rygula, A.; Majzner, K.; Marzec, K.M.; Kaczor, A.; Pilarczyk, M.; Baranska, M. Raman spectroscopy of proteins: a review. *Journal of Raman Spectroscopy* **2013**, *44*, 1061-1076, doi:https://doi.org/10.1002/jrs.4335.
6. Dolui, S.; Mondal, A.; Roy, A.; Pal, U.; Das, S.; Saha, A.; Maiti, N.C. Order, Disorder, and Reorder State of Lysozyme: Aggregation Mechanism by Raman Spectroscopy. *The Journal of Physical Chemistry B* **2020**, *124*, 50-60, doi:10.1021/acs.jpcb.9b09139.
7. Wang, C.-H.; Huang, C.-C.; Lin, L.-L.; Chen, W. The effect of disulfide bonds on protein folding, unfolding, and misfolding investigated by FT–Raman spectroscopy. *Journal of Raman Spectroscopy* **2016**, *47*, 940-947, doi:https://doi.org/10.1002/jrs.4935.
8. Xing, L.; Fan, W.; Chen, N.; Li, M.; Zhou, X.; Liu, S. Amyloid formation kinetics of hen egg white lysozyme under heat and acidic conditions revealed by Raman spectroscopy. *Journal of Raman Spectroscopy* **2019**, *50*, 629-640, doi:https://doi.org/10.1002/jrs.5567.
9. Niaura, G. Raman Spectroscopy in Analysis of Biomolecules. *Encyclopedia of Analytical Chemistry* **2014**, 1-34, doi:https://doi.org/10.1002/9780470027318.a0212.pub3.
